# Predicting the hospitalization burdens of patients with mental disease: a multiple model comparison

**DOI:** 10.3389/fpsyt.2025.1474786

**Published:** 2025-06-18

**Authors:** Lu Hou, Jing Zhang, Li Li, Yelin Weng, Ziyu Yang, Zhiguo Liu

**Affiliations:** ^1^ Department of Information and Statistics Center, Huai’an Third People’s Hospital, Huai’an, China; ^2^ Department of Psychiatry, Huai’an Third People’s Hospital, Huai’an, China; ^3^ Operations Management Department, Huai’an Third People’s Hospital, Huai’an, China; ^4^ College of Intelligent Engineering Technology, Jiangsu Vocational College of Finance and Economics, Huai’an, China

**Keywords:** mental disorder, hospitalization burden, prediction models, time sequence models, regression models

## Abstract

**Background:**

Mental disorders represent a growing public health challenge, with rising hospitalization rates worldwide. Despite their significant impact, systematic investigations into the hospitalization burden (HB) of mental disorders remain notably lacking in current studies.

**Objective:**

This study aims to employ machine learning (ML) techniques to predict the HB among patients with mental disorders. By doing so, we seek to optimize the allocation of medical resources and enhance the efficiency of healthcare services for this specific population.

**Methods:**

Historical hospitalization data were collected, encompassing patient demographics, diagnostic details, length of stay, costs, and other relevant information. The data were then cleaned to remove missing values and outliers, and key features related to the HB were extracted. A statistical analysis of the basic characteristics of the HB was conducted. Subsequently, prediction models for the HB were developed based on the historical data and identified key features, including time series models and regression models. The predictive ability of these models was evaluated by comparing the actual values with the predicted values.

**Results:**

HB was influenced by diagnosis, age, and seasonality, with schizophrenia (A3) and personality disorders (A7) incurring the highest burdens. ML models demonstrated task-specific efficacy: ridge regression for hospitalization frequency, long short-term memory/categorical boosting regression for length of stay, and seasonal autoregressive integrated moving average with exogenous regressors/light gradient boosting machine regression for hospitalization costs. The findings support tailored resource allocation and early intervention for high-risk groups.

**Conclusion:**

This study showcased the effectiveness of machine learning methods in predicting the hospitalization burden of inpatients with mental disorders, thereby offering scientific decision support for medical institutions. This approach contributes to enhancing the quality of patient care and optimizing the efficiency of medical resource utilization.

## Introduction

In contemporary society, mental illness has emerged as a significant issue affecting human health and social wellbeing (1–3). According to the World Mental Health Report (2022), mental disorders affected an estimated 970 million people worldwide, constituting 13% of the global population (4). In Asia alone, 148 million young individuals experience mental disorders, with anxiety and depression showing significant increases (5). These conditions contribute substantially to global disability and premature mortality (6), with prevalence rates rising alongside accelerating life pace, social pressures, and mental health awareness (7, 8). Beyond their health impacts, those with mental disorders face social stigma (9, 10) and mental disorders impose significant burdens on individuals, families, and societies across nations (11).

Inpatient treatment represents a critical component of mental health management (12), with its costs and effectiveness remaining a persistent focus for healthcare policymakers and researchers (13). The burden of hospitalization for patients with mental illness extends beyond the financial implications, encompassing psychological, social, and occupational consequences for both patients and their families (14). A comprehensive understanding of the relationship between mental illness and the burden of hospitalization is essential for optimizing medical resource allocation, improving treatment outcomes, and alleviating societal burdens. Therefore, how to effectively predict and manage the inpatient burden of mental disorder patients has become an urgent issue to be addressed in the healthcare sector.

The rapid advancement of machine learning technology has significantly enhanced its application in the medical field (15, 16), particularly in critical areas such as disease prediction (17), treatment plan optimization (18), and the rational allocation of medical resources. These advanced algorithmic models efficiently process vast and complex medical datasets, accurately uncovering the underlying patterns within them. As a result, they provide clinicians with a scientific basis for understanding disease progression and addressing individualized patient needs. A prediction system for the hospitalization burden of mental disorders, based on machine learning algorithms, can not only improve the accuracy and timeliness of clinical decision-making but also facilitate the optimal allocation of medical resources. This, in turn, effectively reduces the social and economic burdens associated with these conditions.

This study aims to explore and develop a machine learning-based predictive model for assessing the hospitalization burden of patients with mental disorders. We collected and analyzed extensive medical data, including patient demographics, medical history, treatment records, and hospitalization costs. By applying advanced machine learning algorithms, we constructed accurate and efficient predictive models. Through these models, we aspire to forecast the inpatient needs and potential burdens of patients with mental disorders, thereby providing decision support for healthcare institutions and facilitating personalized treatment plans. Ultimately, our goal is to optimize the allocation of medical resources and enhance the quality of life for patients.

## Materials and methods

### Data collection and preprocessing

In the present study, we collected 79,649 hospitalization records of 40,856 inpatients with mental disorders in the Huai’an No.3 People’s Hospital from January 2014 to December 2023. The inclusion criteria were as follows: (1) hospitalized for a psychiatric disorder; (2) hospitalization duration ≥24 hours with complete admission and discharge records; (3) availability of comprehensive clinical data, including demographic characteristics, primary diagnosis, comorbidities, and past medical history. The exclusion criteria were as follows: (1) hospitalization duration <24 hours (e.g., emergency observation cases); (2) not hospitalized for a psychiatric disorder; (3) missing key clinical data, such as admission/discharge time and diagnostic information.

To ensure patient confidentiality, all records underwent de-identification processes, including the encryption of individuals’ national identity numbers. The hospitalization records included demographic information (age and gender), diagnosis results, dates of admission and discharge, and hospitalization costs. During the data preprocessing phase, we eliminated records that contained null or abnormal values, such as when age or total hospitalization cost was erroneously recorded as zero or negative. The special field formats, such as those for age and admission and discharge times, were standardized to ensure uniformity and accuracy across the dataset.

### Definitions for mental disorder classification and hospitalization-related parameters

Age: The age groups of inpatients include four groups: (1) age ≤ 18 years; (2) 18 years < age ≤ 39 years; (3) 39 years < age ≤ 59 years; (4) age ≥ 60 years.

Average hospitalization frequency (AHF): AHF was the ratio of hospitalization frequency (HF) and inpatient number (IN).

Hospitalization costs (HC): the sum of self-pay and medical insurance.

Hospitalization burden (HB): HB consisted of HF, length of stay (LOS), and HC.

Length of stay: LOS was calculated by subtracting the admission date from the discharge date of each admission.

Mental disorder types: The mental disorders of inpatients were divided into 12 types according to the Chinese Classification of Mental Disorders Version 3 (CCMD-3). In order to simplify the expression, the mental disorder types were defined as follows: A1, organic mental disorders; A2, mental disorders due psychoactive substances or non-addictive substances; A3, schizophrenia and other psychotic disorders; A4, mood disorders and affective disorders; A5, hysteria, stress-related disorders, and neurosis; A6, physiological disorders related to psychological factors; A7, personality disorders, habit and impulse disorders, and psychosexual disorders; A8, intellectual disability and disorders of psychological development with onset usually occurring in childhood and adolescence; A9, hyperkinetic, conduct, and emotional disorders with onset usually occurring in childhood and adolescence; A10, other mental disorders and psychological health conditions; A11, patient suffered from two mental disorder types; A12, patient suffered from three or more mental disorder types.

Seasons: Spring, from March to May; Summer, from June to August; Autumn, from September to November; Winter, from December to February of the following year.

### HB prediction models

In this study, the data were collected in the period from 2019 to 2022, a timeframe that coincides with the global COVID-19 pandemic. Recognizing the potential impact of this unprecedented health crisis on mental health services and patient outcomes, we incorporated a novel variable termed the epidemic factor (EF) to account for the presence of COVID-19 effects within the data for each respective year. The EF was defined as a dichotomous variable. Thus, EF=1 indicates cases that either occurred during the COVID-19 pandemic period or were directly impacted by the pandemic, as determined based on the following criteria: a) Time frame from January 2020 to December 2022; b) case characteristics: patients who were either diagnosed with COVID-19 during hospitalization or experienced pandemic-related modifications to medical procedures (e.g., delayed admission and/or canceled surgeries). EF=0 represents cases that either occurred outside the defined pandemic period or showed no direct impact from the pandemic.

This study employed a time-series sliding window approach to partition the dataset, effectively balancing historical information utilization with predictive independence. The temporal division was structured as follows: Training period, January 2014 - December 2020 (84 months); Testing period, January 2021–December 2023 (36 months). For cross-validation, we implemented a sliding window configuration with a window width of 36 months (3 years) and a step size of 12 months (1 year). The data from every four quarters were accumulated to generate annual data to control for seasonal variation.

To comprehensively assess model performance, we employed the following three key metrics: (1) error rate (ER), measuring the relative prediction bias; (2) mean absolute error (MAE), quantifying the absolute prediction accuracy; (3) root mean square error (RMSE), emphasizing larger prediction errors.

#### Time sequence models

Holt’s linear trend model (HLTM) is a statistical method used to predict time sequence data with a linear trend. Its basic idea is to estimate the level and trend of a time sequence using two smoothing equations. The formulas are as follows:


(1) $$L_{t}=\alpha y_{t}+\left(1-\alpha \right)\left(L_{T-1}+T_{t-1}\right)$$

In Equation 1, $L_{t}$ is the level estimate of t , $y_{t}$ is the actual observed value of t , $\alpha \;\left(0\leq \alpha \leq 1\right)$ is the level smoothing coefficient, $L_{t-1}$ is the level estimate of the previous period, and $T_{t-1}$ is the trend estimate of the previous period.


(2) $$T_{t}=\beta \left(L_{t}-L_{t-1}\right)+\left(1-\beta \right)T_{t-1}$$

In Equation 2, $T_{t}$ is the trend estimate of t , $\beta \;\left(0\leq \beta \leq 1\right)$ is the trend smoothing coefficient, $L_{t}-L_{t-1}$ is the value of the current level subtracted from the previous level, and $T_{t-1}$ is the trend estimate of the previous period.


(3) $${\hat{y}}_{t+h}=L_{t}+hT_{t}$$

In Equation 3, ${\hat{y}}_{t+h}$ is the predicted value from t to h , $L_{t}$ is the current level estimate, and $T_{t}$ is the current trend estimate. In common, α and β are estimated by minimizing the prediction error.

Seasonal autoregressive integrated moving average with exogenous regressors (SARIMAX) is an advanced time sequence analysis model that combines seasonal components and exogenous variables to process time series data with seasonal fluctuations and external influences. The SARIMAX model combines five key elements: autoregressive terms, integrated, moving averages of past errors, seasonal patterns, and external variables. The formula for the SARIMAX model is:


(4) $$\phi_{p}\left(L\right){\text{Φ}}_{P}\left(L^{s}\right){\text{Δ}}^{d}{\text{Δ}}_{s}^{D}y_{t}=A\left(t\right)+\theta_{q}\left(L\right){\text{Θ}}_{Q}\left(L^{s}\right)\epsilon_{t}$$

In Equation 4, $\phi_{p}\left(L\right)$ and ${\text{Φ}}_{P}\left(L^{s}\right)$ are the polynomials of the autoregressive and seasonal autoregressive, respectively; $\theta_{q}\left(L\right)$ and ${\text{Θ}}_{Q}\left(L^{s}\right)$ are the polynomials of the moving average and the seasonal moving average, respectively; $\Delta^{d}$ and $\Delta_{s}^{D}$ are the difference operators for the non-seasonal and seasonal, respectively; $y_{t}$ is time sequence data; $A\left(t\right)$ is the linear combination of exogenous variables; and $\epsilon_{t}$ is the white noise error term. Parameter estimation in the SARIMAX model is usually performed by maximum likelihood estimation or the conditional least square method.

Long short-term memory (LSTM) is a special recurrent neural network (RNN) architecture, and its key units include a cell state, input gate, forget gate, and output gate. The formulas of the key units are as follows.

Forget gate:


(5) $$f_{t}=\sigma \left(W_{f}\cdot \left[h_{t-1},x_{t}\right]+b_{f}\right)$$

In Equation 5, $f_{t}$ is the output of the forget gate; σ is the sigmoid function; $W_{f}$ and $b_{f}$ are the weight matrix and the bias vector, respectively; $h_{t-1}$ is the hidden state of the previous moment; and $x_{t}$ is the input to the current moment.

Input gate:


(6) $$i_{t}=\sigma \left(W_{i}\cdot \left[h_{t-1},x_{t}\right]+b_{i}\right)$$

(7) $${\tilde{C}}_{t}=\text{tanh}\left(W_{C}\cdot \left[h_{t-1},x_{t}\right]+b_{c}\right)$$

In Equations 6 and 7, $i_{t}$ is the output of the input gate, ${\tilde{C}}_{t}$ is the new candidate values vector, and tanh is the hyperbolic tangent function.

Update cell state:


(8) $$C_{t}=f_{t}\cdot C_{t-1}+i_{t}\cdot {\tilde{C}}_{t}$$

In Equation 8, *C_t_* is the current state of the cell.

Output gate:


(9) $$o_{t}=\sigma \left(W_{o}\cdot \left[h_{t-1},x_{t}\right]+b_{o}\right)$$

(10) $$h_{t}=o_{t}\cdot \text{tanh}\left(C_{t}\right)$$

In Equations 9 and 10, *o_t_* is the output of the output gate and *h_t_* is the hidden state of the current moment.

The LSTM model, through the combination of these gates, is able to learn when to forget the old information, when to read the new input, and when to update the state of the memory cells, so as to effectively deal with long sequences depending on the problem.

#### Regression models

Ridge regression (RR) is a regularization method for linear regression that aims to solve multicollinearity and overfitting problems. It does this by adding an L2 regularization term, the sum of squares of the weights, to the loss function. We used an RR model (RidgeCV), through cross validation, to select the optimal regularization parameter α value.

Light gradient boosting machine regression (LGBMR) is a gradient boosting framework based on the decision tree algorithm, and it is able to process large-scale data efficiently and quickly. The key parameters are set as follows: Num_leaves, 31; max_depth, -1; learning_rate, 0.1; n_estimators, 100.

Categorical boosting regression (CBR) is based on the gradient of decision tree machine learning algorithms, and it is especially suitable for processing that includes classification variable datasets. The key parameters are set as follows: iterations, 1,000; learning_rate, 0.03; depth, 6; L2_leaf_reg, 3.0.

### Statistical analysis

Statistical descriptions and analyses were performed for season, gender, age, and disease distribution characteristics of the hospitalized patients. The statistical analysis methods used included the chi-square test, the normality and lognormality tests, and ANOVA analysis. A *P*-value less than 0.05 indicated a significant difference.

## Results

### The baseline characteristics of the inpatients with mental disorders

**Figure 1:**
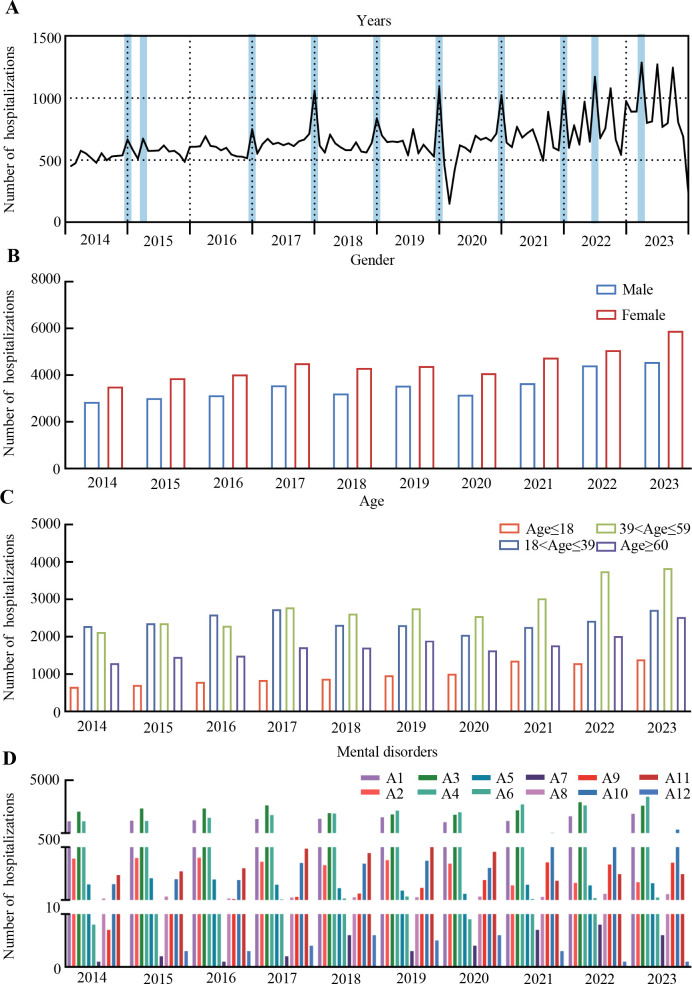
The number of hospitalizations from 2014 to 2023. **(A)** Monthly number of hospitalizations from 2014 to 2023, with the largest hospitalization number in each year highlighted in blue. **(B)** Number of hospitalizations of men and women. **(C)** Number of hospitalizations among different age ranges. **(D)** Number of hospitalizations of patients with different mental disorder types.

**Figure 1A** shows the HF in different months from 2014 to 2023. The blue backgrounds highlight the largest HF in each year. For most years, the HF in December was the largest. Notably, there was a significant decrease in hospitalizations at the beginning of 2020. This phenomenon was attributed to the outbreak of the COVID-19 pandemic. The pandemic led to significant changes in healthcare utilization patterns, with many individuals potentially avoiding hospitals due to fear of infection, lockdowns, or restrictions on non-essential medical services. This could have resulted in a reduction in routine hospital admissions and affected the hospitalization rates. Further analysis would be needed to confirm this correlation. Moreover, the number of female inpatients was significantly higher than that of male inpatients (**Figure 1B**). The HF in different age ranges had obvious differences (**Figure 1C**). Patients with organic mental disorders (A1), schizophrenia and other psychotic disorders (A3), and mood disorders and affective disorders (A4) comprised most of the total hospital admissions (**Figure 1D**).

### HB of inpatients with mental disorders

#### AHF

During the last decade, the HF and IN showed a trend of first increasing, then decreasing, and then increasing again. In brief, from 2014 to 2017, the HF increased and the IN increased too. During this period, the AHF also showed an increasing trend. In 2018, the HF decreased, but the IN increased, while the AHF decreased. In 2020, the values of HF, IN, and AHF all reached their lowest points in the past decade. This may be due to the lockdown measures implemented at the beginning of the COVID-19 pandemic. However, as the pandemic progressed and the duration of lockdown measures extended, people’s mental health was severely impacted. In 2021–2022, despite the ongoing lockdown measures, the HF, IN, and AHF for patients with mental disorders increased (**Supplementary Table 1**).

Furthermore, the AHF of different mental disorder types was analyzed. Thereinto, personality disorders, habit and impulse disorders, and psychosexual disorders (A7) had the largest AHF among the mental disorder types, followed by schizophrenia and other psychotic disorders (A3) and patients suffered from two types of mental disorder (A11). Physiological disorders related to psychological factors (A6) had the lowest AHF (**Supplementary Table 2**).

The AHF in different seasons was further analyzed, and the results showed that summer had the largest AHF while winter had the lowest AHF in a year (**Supplementary Table 3**).

#### LOS

According to the data distribution, the LOS of inpatients was divided into four ranges. Those with a LOS of less than 30 days had the highest HF, while as the LOS increased, HF showed a decreasing trend (**Supplementary Table 4**).

Patients with personality disorders, habit and impulse disorders, or psychosexual disorders (A7) had the largest LOS, while those with intellectual disability and disorders of psychological development with onset usually occurring in childhood and adolescence (A8) and those with schizophrenia and other psychotic disorders (A3) had the second and third LOS, respectively. Patients with physiological disorders related to psychological factors (A6) had the shortest LOS (**Supplementary Table 5**).

Patients hospitalized in the winter had the longest LOS, while those hospitalized in spring had the shortest LOS (**Supplementary Table 6**).

#### HC

Those with an HC in the ranges of 5,000–10,000 and 10,000–20,000 had higher HF than those with an HC lower than 5,000 or higher than 20,000 (**Supplementary Table 7**).

Patients with personality disorders, habit and impulse disorders, or psychosexual disorders (A7) had the largest HC among the mental disorder types, followed by those with schizophrenia and other psychotic disorders (A3) and patients who suffered from two types of mental disorder (A11). Those with physiological disorders related to psychological factors (A6) had the lowest HC (**Supplementary Table 8**).

Among the four seasons, winter had the highest HC (**Supplementary Table 9**).

### The prediction of HB for patients with mental disorders

**Figure 2:**
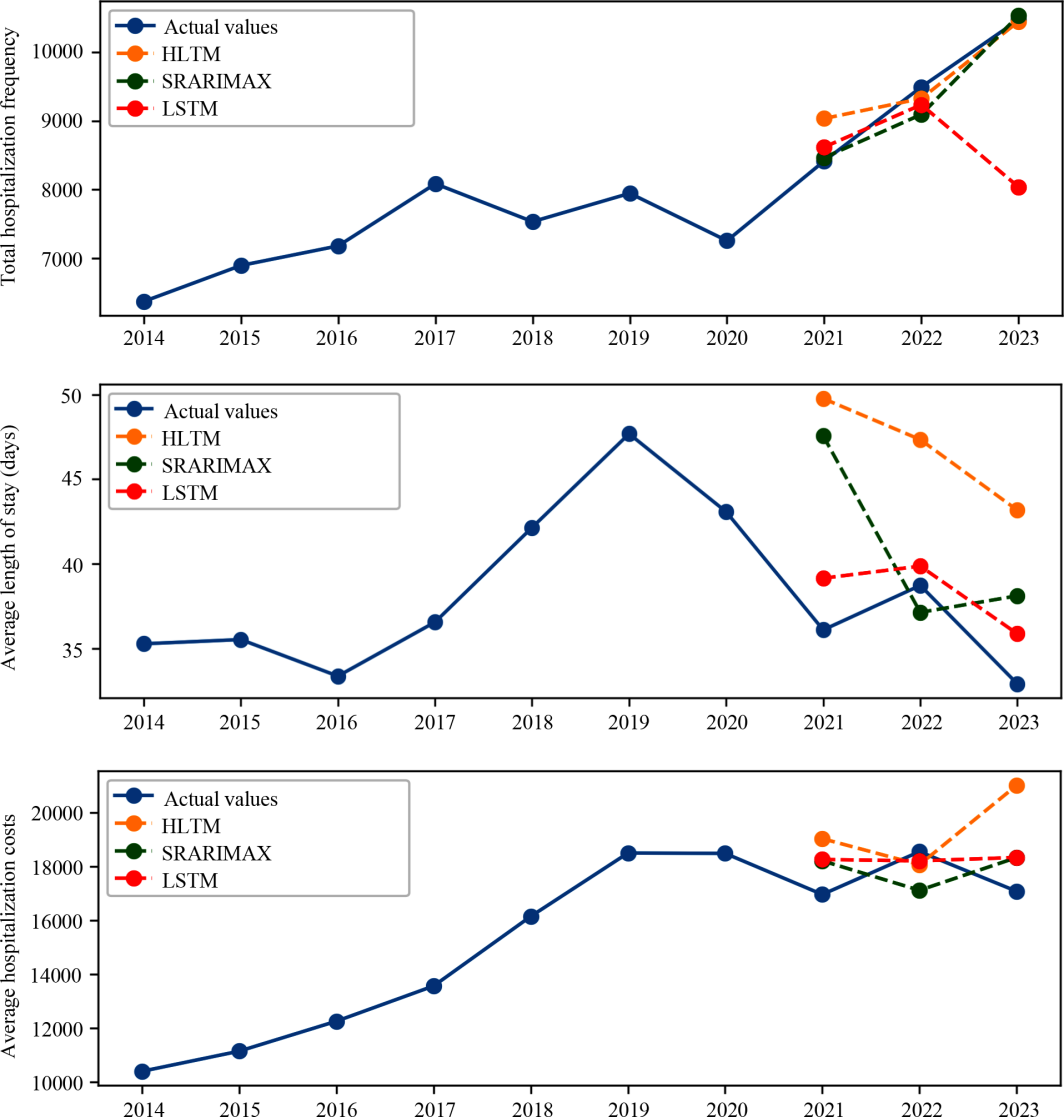
Time sequence models predicted the hospitalization burden (HB) of patients with mental disorders. The HLTM, SARIMAX, and LSTM models predicted the hospitalization frequency (HF) (top), length of stay (LOS) (middle), and hospitalization cost (HC) (bottom).

**Figure 3:**
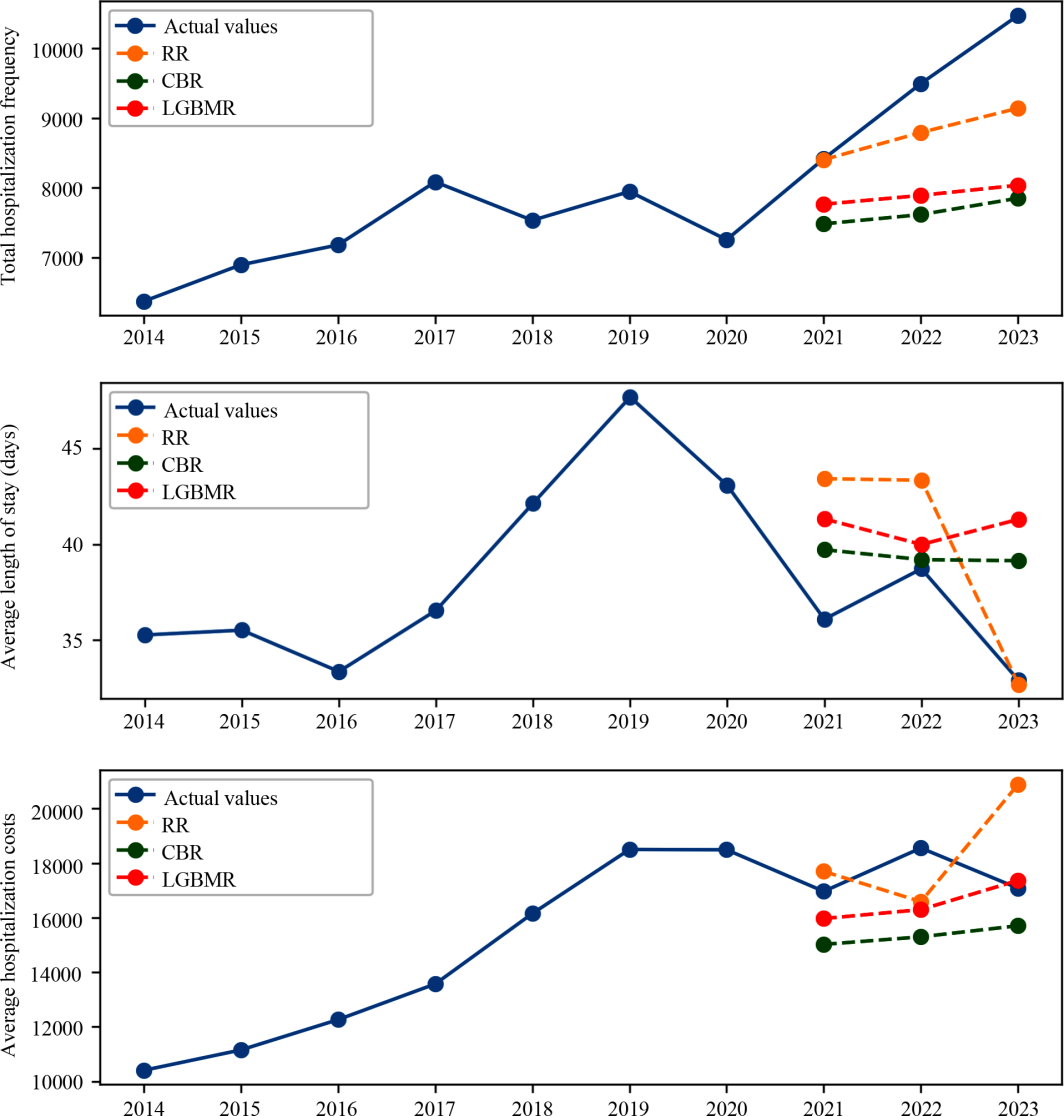
Regression models predicted the hospitalization burden (HB) of patients with mental disorders. The RR, CBR and LGBMR models predicted the hospitalization frequency (HF) (top), length of stay (LOS) (middle), and hospitalization cost (HC) (bottom).

In the current study, we used time sequence models and regression models to predict the HB in 2021, 2022, and 2023. Furthermore, we compared the predicted values (PVs) with the actual values (AVs) and introduced the error rate (ER) to test the predictive abilities of the models. Among the models, we found three models with better predictive performance among the time sequence models and regression models, respectively (**Figures 2**, **3** and **Tables 1**–**3**). The results of other models are shown in **Supplementary Table 10**.

**Table 1:** True and predicted values of hospitalization frequency predicted by the time sequence (top) and regression (middle) models, as well as evaluation of the predictive performance of the models (bottom).

HF	HLTM		SARIMAX		LSTM	
Year	TV	PV	TV	PV	TV	PV
2021	8416	9034.99	8416	8466.42	8416	8619.17
2022	9495	9327.09	9495	9094.58	9495	9235.82
2023	10473	10449.21	10473	10536.48	10473	10109.82
HF	RR		CBR		LGBMR	
Year	TV	PV	TV	PV	TV	PV
2021	8416	8403.10	8416	7482.33	8416	7763.76
2022	9495	8793.72	9495	7615.71	9495	7890.96
2023	10473	1331.41	10473	7850.63	10473	8036.91
Models	MAE		RMSE		ER	
HLTM	270.23		370.57		3.12	
SARIMAX	171.44		235.87		1.81	
LSTM	275.18		283.04		2.87	
RR	3285.26		5293.77		6.75	
CBR	1811.78		798.46		18.64	
LGBMR	1564.12		652.22		15.97	

HF, hospitalization frequency; HLTM, Holt’s linear trend model; SARIMAX, Seasonal autoregressive integrated moving average with exogenous regressors; LSTM, Long short-term memory; RR, Ridge regression; CBR, Categorical Boosting regression; LGBMR, Light gradient boosting machine regression; MAE, Mean Absolute Error; RMSE, Root Mean Square Error; ER, Error Rate; TV, True values; PV, Predicted values.

**Table 2:** True and predicted values of length of stay predicted by the time sequence (top) and regression (middle) models, as well as evaluation of the predictive performance of the models (bottom).

LOS	HLTM		SARIMAX		LSTM	
Year	TV	PV	TV	PV	TV	PV
2021	36.09	49.77	36.09	47.56	36.09	39.15
2022	38.72	47.34	38.72	37.13	38.72	39.86
2023	32.90	43.17	32.90	38.11	32.90	35.86
LOS	RR		CBR		LGBMR	
Year	TV	PV	TV	PV	TV	PV
2021	36.09	43.42	36.09	39.72	36.09	41.32
2022	38.72	43.34	38.72	39.20	38.72	39.99
2023	32.90	32.69	32.90	39.14	32.90	41.30
Models	MAE		RMSE		ER	
HLTM	10.86		11.04		30.44	
SARIMAX	6.09		7.33		17.24	
LSTM	2.39		2.54		6.79	
RR	4.05		5.00		10.96	
CBR	3.45		4.18		10.08	
LGBMR	4.97		5.76		10.27	

LOS, Length of stay; HLTM, Holt’s linear trend model; SARIMAX, Seasonal autoregressive integrated moving average with exogenous regressors; LSTM, Long short-term memory; RR, Ridge regression; CBR, Categorical Boosting regression; LGBMR, Light gradient boosting machine regression; MAE, Mean Absolute Error; RMSE, Root Mean Square Error; ER, Error Rate; TV, True values; PV, Predicted values.

**Table 3:** True and predicted values of hospitalization cost predicted by the time sequence (top) and regression (middle) models, as well as evaluation of the predictive performance of the models (bottom).

HC	HLTM		SARIMAX		LSTM	
Year	TV	PV	TV	PV	TV	PV
2021	16,965.29	19,036.08	16,965.29	18,214.44	16,965.29	18,262.53
2022	18,554.55	19,069.78	18,554.55	17,119.28	18,554.55	18,211.99
2023	17,081.18	21,016.82	17,081.18	18,336.94	17,081.18	18,339.02
HC	RR		CBR		LGBMR	
Year	TV	PV	TV	PV	TV	PV
2021	16965.29	17696.75	16965.29	15021.57	16965.29	15968.54
2022	18554.55	16588.22	18554.55	15299.25	18554.55	16297.74
2023	17081.18	20887.31	17081.18	15706.16	17081.18	17366.96
Models	MAE		RMSE		ER	
HLTM	2173.89		2583.54		12.62	
SARIMAX	1313.39		1316.22		7.48	
LSTM	965.88		1062.79		5.62	
RR	2167.97		2509.21		12.40	
CBR	2191.35		2328.90		12.35	
LGBMR	1179.78		1433.90		6.57	

HC, hospitalization cost; HLTM, Holt’s linear trend model; SARIMAX, Seasonal autoregressive integrated moving average with exogenous regressors; LSTM, Long short-term memory; RR, Ridge regression; CBR, Categorical Boosting regression; LGBMR, Light gradient boosting machine regression; MAE, Mean Absolute Error; RMSE, Root Mean Square Error; ER, Error Rate; TV, True values; PV, Predicted values.

Among the time sequence models, SARIMAX and HLTM models showed higher accuracy when predicting HF. The SARIMAX and LSTM models had more advantages in predicting HC. The LSTM model was able to predict LOS more accurately (**Figure 2** and the top parts of **Tables 1**-**3**). In order to improve the performance of the prediction models, regression models were applied and more inpatient characteristics were incorporated into the models. The inpatient characteristics are displayed in **Supplementary Table 11**. In this part, the RR models showed a lower ER in HF prediction. For LOS prediction, the CBR and RR models had more accurate predictions, and the CBR models showed more stable predictive performance. The RR and LGBM models had better prediction performance for HC (**Figure 3** and the bottom parts of **Tables 1**-**3**).

## Discussion

In the current study, we first analyzed the basic characteristics of inpatients with mental disorders and found that factors such as month, gender, age, and types of mental disorders influenced HF. To address the gap in research concerning HB for patients with mental disorders, we further evaluated the AHF, LOS, and HC of these inpatients. Our results indicated that HB was associated with both the type of mental disorder and seasonal variations. Furthermore, we employed various time series models and regression models to predict HB, and these predictive models demonstrated strong performance in forecasting HB.

Epidemiological evidence indicates that women have a significantly higher prevalence of depression and post-traumatic stress disorder (PTSD) compared to men (19–21). This disparity may be attributed to neuroendocrine differences, such as HPA axis regulation (22); psychosocial factors, including gender role stress; and variations in drug metabolism, for example, CYP2C19 polymorphism (23). Our study further revealed that the proportion of female inpatients was notably higher at 63.2%, suggesting a greater disease burden and potential differences in treatment responses. Future research should focus on optimizing gender-specific intervention strategies, including synchronized hormone therapy, cognitive reinforcement for trauma, and individualized dosing regimens.

Age plays a crucial role in the stratification of the onset and clinical phenotypes of mental disorders. In childhood and adolescence, neurodevelopmental disorders, such as attention-deficit/hyperactivity disorder (ADHD) (24), depressive disorders (25), and social anxiety disorders (26). Young adulthood (ages 15–30) marks a high-risk period for bipolar disorder (27) and schizophrenia (28), with 70% of first episodes occurring during this time. This phenomenon may be linked to delayed myelination in the prefrontal cortex. In middle age (ages 35–55), people may be at risk for depression, anxiety (29), and substance use disorders (30). Furthermore, depression (31) and anxiety in later life have been identified as predictors of cognitive decline (32).

In this study, we found that young and middle-aged patients exhibited a higher HF, while children and adolescents had the lowest HF. Our findings also indicated that patients with organic mental disorders (A1), schizophrenia and other psychotic disorders (A3), and mood and affective disorders (A4) experienced higher HF compared to those with other types of mental disorders. We hypothesize that young and middle-aged patients may be more susceptible to severe mental disorders, such as major depression, bipolar disorder, and schizophrenia, which may require hospitalization to ensure patient safety and facilitate appropriate treatment. This vulnerability could be a key factor influencing the decision of young and middle-aged patients to seek hospitalization.

In the existing research, there were limited numbers of studies on the inpatient burden of patients with mental disorders, thus, we further analyzed the HB of inpatients with mental disorders and filled the gap. For mental disorder types, patients with schizophrenia and other psychotic disorders (A3) and personality disorders, habit and impulse disorders, or psychosexual disorders (A7) suffered from higher HB, such as more AHF, longer LOS, and higher HC. However, patients with physiological disorders related to psychological factors (A6) suffered from a lower HB. The rising burden of schizophrenia has emerged as a global concern (33). Factors such as the lack of effective treatment options and a high relapse rate contribute to the significant hospitalization burden associated with this disorder. Personality disorders face similar challenges (34).

Moreover, the current study used many time sequence models and regression models to predict the HB and these models showed good predictive abilities. When constructing the time sequence model to predict the HF, we found that the predictive model efficacy was poor. To improve the predictive performance of our model during the COVID-19 pandemic from late 2019 to late 2022, we introduced a new variable to characterize the impact of the pandemic. This variable took into account the disruption to medical care caused by the pandemic. Additionally, we segmented the data by seasons to account for the seasonal variations in hospitalizations. By incorporating these adjustments, we aimed to enhance the model’s ability to accurately predict HF during this unique period (**Supplementary Table 12**).

Among the time series forecasts, the SARIMAX model showed higher stability in HF and HC forecasts thanks to its ability to model seasonal trends and exogenous variables (e.g., epidemics and policy changes), allowing it to more reliably capture the impact of external shocks. In contrast, the LSTM model has advantages in LOS prediction due to its learning ability of long-term dependencies, which is especially suitable for extracting complex time patterns from historical data to improve prediction accuracy.

In the medical prediction task, different models exhibited optimized performance for specific metrics. The impressive performance of the RR model in predicting HF can be largely attributed to its regularization mechanism, specifically the L2 penalty term. This feature effectively addresses multicollinearity issues, such as the high correlation between health indicators and medical records, thereby enhancing prediction stability and generalization capability. In contrast, LGBMR and CBR demonstrated significant advantages in predicting LOS and HC. Their decision tree-based ensemble learning frameworks enable efficient processing of high-dimensional data, automatic feature binning, and the modeling of non-linear relationships. This makes them particularly well-suited for analyzing large-scale medical datasets.

In this study, we have elucidated the hospitalization burden and its characteristics among patients with mental disorders, filling a gap in the research on the hospitalization burden of mental disorders. Based on this, we have established time series models and regression models for predicting hospitalization burden. We selected the optimal predictive model for forecasting, tailored to the specific types of hospitalization burden. In a future study, we need to combine the time sequence model with the regression model to further provide the predictive performance of the model. Early prediction of hospitalization burden is beneficial for the rational allocation of medical resources in hospitals and enhances the understanding of the societal and familial burden of mental disorders.

## Data Availability

The raw data supporting the conclusions of this article will be made available by the authors, without undue reservation.
