# Supplementary material for: Predicting the hospitalization burdens of patients with mental disease: a multiple model comparison
Source: Front Psychiatry. 2025 Jun 18;16:1474786. doi: 10.3389/fpsyt.2025.1474786 (PMC12216087; doi:10.3389/fpsyt.2025.1474786)
Supplement: Supplementary file 1 [file Table1.docx]

**Supplementary Table 1. Number of hospitalizations from 2014 to 2023.**

| Year | Frequency of hospitalizations | Number of inpatients | AFH |
| --- | --- | --- | --- |
| 2014 | 6371 | 5021 | 1.27 |
| 2015 | 6895 | 5349 | 1.29 |
| 2016 | 7180 | 5259 | 1.37 |
| 2017 | 8085 | 5803 | 1.39 |
| 2018 | 7533 | 6090 | 1.24 |
| 2019 | 7946 | 6389 | 1.24 |
| 2020 | 7255 | 6092 | 1.19 |
| 2021 | 8416 | 6759 | 1.25 |
| 2022 | 9495 | 7032 | 1.35 |
| 2023 | 10473 | 8277 | 1.27 |
| Average | 7964.90 | 6207.10 | 1.29 |

**Supplementary Table 2. Average number of hospitalizations of different mental disorder types from 2014 to 2023.**

| Year | A1 | A2 | A3 | A4 | A5 | A6 | A7 | A8 | A9 | A10 | A11 | A12 |
| --- | --- | --- | --- | --- | --- | --- | --- | --- | --- | --- | --- | --- |
| 2014 | 1.54 | 1.53 | 1.66 | 1.63 | 1.32 | 1.00 | 1.00 | 1.28 | 1.14 | 1.32 | 1.57 | 0.00 |
| 2015 | 1.46 | 1.70 | 1.84 | 1.59 | 1.43 | 1.80 | 2.00 | 1.38 | 1.00 | 1.44 | 1.80 | 1.33 |
| 2016 | 1.46 | 1.60 | 2.17 | 1.72 | 1.68 | 1.18 | 1.00 | 1.46 | 1.20 | 1.55 | 1.92 | 1.67 |
| 2017 | 1.56 | 1.51 | 2.36 | 1.62 | 1.62 | 1.31 | 3.00 | 1.76 | 1.66 | 1.52 | 2.15 | 1.50 |
| 2018 | 1.42 | 1.65 | 1.71 | 1.45 | 1.27 | 1.08 | 2.00 | 1.30 | 1.06 | 1.38 | 1.62 | 1.17 |
| 2019 | 1.48 | 1.69 | 1.75 | 1.46 | 1.10 | 1.05 | 2.33 | 1.37 | 1.12 | 1.38 | 1.70 | 1.80 |
| 2020 | 1.27 | 1.46 | 1.59 | 1.42 | 1.06 | 1.00 | 1.50 | 1.26 | 1.08 | 1.17 | 1.66 | 1.33 |
| 2021 | 1.39 | 1.48 | 1.78 | 1.49 | 1.21 | 1.15 | 2.43 | 2.05 | 1.19 | 1.42 | 1.97 | 1.00 |
| 2022 | 1.48 | 1.92 | 2.52 | 1.51 | 1.21 | 1.10 | 4.00 | 1.96 | 1.24 | 1.32 | 2.58 | 2.00 |
| 2023 | 1.38 | 2.12 | 1.99 | 1.45 | 1.20 | 1.09 | 3.00 | 1.48 | 1.17 | 1.40 | 1.94 | 3.00 |
| Average | 1.44 | 1.67 | 1.94 | 1.53 | 1.31 | 1.18 | 2.23 | 1.53 | 1.19 | 1.39 | 1.89 | 1.48 |

Since some data groups violated the normality assumption (A6, A8 and A9), nonparametric analysis was performed using the Kruskal-Wallis rank sum test, which showed statistically significant differences (H=59.24, P < 0.001).

**Supplementary Table 3. Average number of hospitalizations of different seasons from 2014 to 2023.**

| Year | Spring | Summer | Autumn | Winter |
| --- | --- | --- | --- | --- |
| 2014 | 1.10 | 1.09 | 1.12 | NA |
| 2015 | 1.13 | 1.13 | 1.13 | 1.04 |
| 2016 | 1.19 | 1.18 | 1.17 | 1.07 |
| 2017 | 1.14 | 1.18 | 1.18 | 1.06 |
| 2018 | 1.06 | 1.08 | 1.06 | 1.11 |
| 2019 | 1.06 | 1.05 | 1.04 | 1.03 |
| 2020 | 1.04 | 1.05 | 1.06 | 1.03 |
| 2021 | 1.06 | 1.06 | 1.05 | 1.02 |
| 2022 | 1.03 | 1.04 | 1.04 | 1.02 |
| 2023 | 1.04 | 1.65 | 1.04 | 1.03 |
| Average | 1.08 | 1.15 | 1.09 | 1.05 |

Since some data groups violated the normality assumption (summer, autumn and winter), nonparametric analysis was performed using the Kruskal-Wallis rank sum test, which showed no statistically significant differences (H=7.62, P = 0.055).

**Supplementary Table 4. Length of stay from 2014 to 2023.**

| Year | <30 days | 1. days-90 days | 1. days-180days | >180 days |
| --- | --- | --- | --- | --- |
| 2014 | 4498 | 1509 | 144 | 219 |
| 2015 | 4991 | 1483 | 197 | 224 |
| 2016 | 5431 | 1345 | 180 | 224 |
| 2017 | 5886 | 1664 | 213 | 322 |
| 2018 | 4595 | 2326 | 269 | 343 |
| 2019 | 4948 | 2027 | 405 | 566 |
| 2020 | 4658 | 1758 | 392 | 447 |
| 2021 | 5611 | 1797 | 886 | 122 |
| 2022 | 5585 | 2716 | 1098 | 96 |
| 2023 | 7002 | 2080 | 1329 | 62 |
| Average | 5320.5 | 1870.50 | 511.30 | 262.5 |

Since some data groups violated the normality assumption (90-180 days), nonparametric analysis was performed using the Kruskal-Wallis rank sum test, which showed statistically significant differences (H=33.22, P < 0.001).

**Supplementary Table 5-1. Length of stay of different mental disorder types from 2014 to 2023.**

| Year | A1 | A2 | A3 | A4 | A5 | A6 |
| --- | --- | --- | --- | --- | --- | --- |
| 2014 | 20.23 | 16.91 | 53.59 | 24.85 | 16.61 | 11.88 |
| 2015 | 17.29 | 16.74 | 54.54 | 25.17 | 18.20 | 10.20 |
| 2016 | 16.85 | 17.10 | 50.40 | 23.84 | 19.60 | 7.45 |
| 2017 | 18.59 | 18.67 | 56.14 | 24.21 | 20.27 | 12.56 |
| 2018 | 21.49 | 20.53 | 73.78 | 28.45 | 25.86 | 17.12 |
| 2019 | 22.92 | 25.07 | 92.65 | 28.23 | 20.98 | 16.81 |
| 2020 | 20.98 | 22.38 | 87.97 | 24.41 | 18.52 | 15.44 |
| 2021 | 17.80 | 41.59 | 69.61 | 22.16 | 21.34 | 14.05 |
| 2022 | 18.57 | 40.32 | 68.68 | 23.99 | 23.97 | 9.80 |
| 2023 | 16.14 | 36.54 | 63.00 | 22.77 | 23.92 | 9.29 |
| Average | 19.09 | 25.59 | 67.04 | 24.81 | 20.93 | 12.46 |

**Supplementary Table 5-2. Length of stay of different mental disorder types from 2014 to 2023.**

| Year | A7 | A8 | A9 | A10 | A11 | A12 |
| --- | --- | --- | --- | --- | --- | --- |
| 2014 | 0.00 | 75.60 | 17.86 | 20.18 | 66.16 | 0.00 |
| 2015 | 60.50 | 35.90 | 19.30 | 24.02 | 70.17 | 13.33 |
| 2016 | 90.00 | 72.73 | 18.10 | 20.05 | 67.22 | 36.33 |
| 2017 | 31.00 | 133.00 | 18.93 | 20.16 | 62.26 | 21.00 |
| 2018 | 130.33 | 98.54 | 22.77 | 23.47 | 65.24 | 73.00 |
| 2019 | 248.67 | 117.68 | 21.31 | 24.98 | 83.67 | 103.00 |
| 2020 | 117.75 | 48.74 | 17.57 | 19.05 | 46.83 | 64.67 |
| 2021 | 132.14 | 49.15 | 17.24 | 17.19 | 61.97 | 28.67 |
| 2022 | 83.88 | 55.19 | 17.77 | 17.52 | 64.31 | 115.00 |
| 2023 | 89.83 | 41.00 | 17.34 | 17.12 | 58.74 | 90.00 |
| Average | 98.41 | 72.75 | 18.82 | 20.37 | 64.66 | 54.50 |

Since some data groups violated the normality assumption (A2 and A9), nonparametric analysis was performed using the Kruskal-Wallis rank sum test, which showed statistically significant differences (H=80.83, P < 0.001).

**Supplementary Table 6. Length of stay of different seasons from 2014 to 2023.**

| Year | Spring | Summer | Autumn | Winter |
| --- | --- | --- | --- | --- |
| 2014 | 26.93 | 25.77 | 29.44 | NA |
| 2015 | 27.43 | 32.85 | 29.19 | 54.02 |
| 2016 | 25.45 | 25.40 | 26.33 | 50.15 |
| 2017 | 27.92 | 25.18 | 31.14 | 55.49 |
| 2018 | 33.02 | 30.78 | 42.42 | 60.20 |
| 2019 | 30.20 | 38.14 | 27.30 | 63.46 |
| 2020 | 32.25 | 36.27 | 27.19 | 98.28 |
| 2021 | 28.39 | 35.19 | 36.29 | 65.27 |
| 2022 | 36.41 | 39.25 | 37.54 | 43.16 |
| 2023 | 35.16 | 37.12 | 32.11 | 37.92 |
| Average | 30.32 | 32.59 | 31.89 | 58.66 |

Parametric analysis was performed using one-way ANOVA as the data satisfied normality assumptions, revealing statistically significant differences among groups (F=17.14, P< 0.001).

**Supplementary Table 7. Hospitalization costs from 2014 to 2023.**

| Year | <5,000 | 5,000–10,000 | 10,000-20,000 | >20,000 |
| --- | --- | --- | --- | --- |
| 2014 | 1406 | 3308 | 1159 | 497 |
| 2015 | 1311 | 3578 | 1382 | 624 |
| 2016 | 1195 | 3307 | 1984 | 694 |
| 2017 | 1251 | 3125 | 2790 | 919 |
| 2018 | 1075 | 1770 | 3248 | 1440 |
| 2019 | 1062 | 2010 | 2917 | 1957 |
| 2020 | 859 | 1845 | 2536 | 2015 |
| 2021 | 957 | 2412 | 2817 | 2230 |
| 2022 | 1051 | 2024 | 2906 | 3514 |
| 2023 | 1205 | 2339 | 3697 | 3232 |
| Average | 1137.20 | 2571.80 | 2543.60 | 1712.20 |

Parametric analysis was performed using one-way ANOVA as the data satisfied normality assumptions, revealing statistically significant differences among groups (F=8.40, P< 0.001).

**Supplementary Table 8-1. Hospitalization costs of different mental disorder types from 2014 to 2023.**

| Year | A1 | A2 | A3 | A4 | A5 | A6 |
| --- | --- | --- | --- | --- | --- | --- |
| 2014 | 7530.14 | 8120.63 | 13368.09 | 8744.69 | 6912.27 | 5785.28 |
| 2015 | 7067.48 | 8059.64 | 14536.30 | 9686.66 | 8091.24 | 4715.95 |
| 2016 | 7929.91 | 8806.22 | 16266.12 | 10450.17 | 8465.52 | 4732.81 |
| 2017 | 8668.44 | 9662.48 | 18337.88 | 10993.85 | 10042.23 | 7547.04 |
| 2018 | 9624.15 | 10904.83 | 24470.34 | 13465.17 | 13036.26 | 8248.90 |
| 2019 | 10460.64 | 12531.63 | 31230.53 | 13985.06 | 12223.42 | 8832.34 |
| 2020 | 10108.45 | 11840.56 | 32799.43 | 13431.19 | 11269.58 | 9998.33 |
| 2021 | 9347.49 | 19246.77 | 29510.37 | 12276.64 | 12526.47 | 7453.71 |
| 2022 | 10285.71 | 19350.98 | 29378.84 | 14016.86 | 14937.02 | 7213.17 |
| 2023 | 9399.61 | 17751.27 | 28409.76 | 14269.58 | 15664.53 | 6475.46 |
| Average | 9042.20 | 12627.50 | 23830.77 | 12131.99 | 11316.85 | 7100.30 |

**Supplementary Table 8-2. Hospitalization costs of different mental disorder types from 2014 to 2023.**

| Year | A7 | A8 | A9 | A10 | A11 | A12 |
| --- | --- | --- | --- | --- | --- | --- |
| 2014 | 852.80 | 15114.62 | 5779.96 | 7798.34 | 17580.77 | 0.00 |
| 2015 | 10837.59 | 10257.75 | 6343.32 | 8980.92 | 20071.94 | 4593.32 |
| 2016 | 30095.39 | 16080.95 | 7981.80 | 8946.85 | 20659.17 | 11998.19 |
| 2017 | 9604.48 | 32409.14 | 8326.60 | 9287.36 | 20066.04 | 9100.96 |
| 2018 | 34648.43 | 19841.07 | 10467.70 | 10826.04 | 22848.26 | 23002.46 |
| 2019 | 68704.38 | 27317.55 | 10337.31 | 10953.10 | 29170.07 | 33209.30 |
| 2020 | 36902.19 | 18714.09 | 9813.54 | 10088.17 | 20352.63 | 24021.68 |
| 2021 | 50620.18 | 21722.11 | 9888.54 | 9338.07 | 25607.37 | 14670.76 |
| 2022 | 30675.55 | 22228.11 | 10817.91 | 10298.05 | 27532.15 | 55678.39 |
| 2023 | 36973.65 | 17543.48 | 10732.01 | 10935.44 | 25676.77 | 42424.09 |
| Average | 30991.46 | 20122.89 | 9048.87 | 9745.24 | 22956.52 | 21869.91 |

Since some data groups violated the normality assumption (A2), nonparametric analysis was performed using the Kruskal-Wallis rank sum test, which showed statistically significant differences (H=66.67, P < 0.001).

**Supplementary Table 9. Hospitalization costs of different seasons from 2014 to 2023.**

| Year | Spring | Summer | Autumn | Winter |
| --- | --- | --- | --- | --- |
| 2014 | 8763.20 | 8726.31 | 9245.78 | NA |
| 2015 | 9503.24 | 10197.84 | 9745.01 | 14570.41 |
| 2016 | 10436.70 | 10493.91 | 10830.58 | 15736.42 |
| 2017 | 11923.98 | 10781.15 | 11476.19 | 17734.95 |
| 2018 | 14164.42 | 13678.77 | 14681.61 | 20263.77 |
| 2019 | 14214.94 | 16013.31 | 13636.73 | 22584.64 |
| 2020 | 15977.98 | 16283.52 | 14380.42 | 31306.02 |
| 2021 | 14426.23 | 15762.40 | 17300.95 | 24348.51 |
| 2022 | 17852.74 | 18516.83 | 18343.56 | 20442.81 |
| 2023 | 17996.70 | 18541.48 | 16106.40 | 18818.15 |
| Average | 13526.01 | 13899.55 | 13574.72 | 20645.07 |

Parametric analysis was performed using one-way ANOVA as the data satisfied normality assumptions, revealing statistically significant differences among groups (F=6.92, P=0.001).

**Supplementary table 10. Other regression models predicted the hospitalization burdens.**

| HF |  | ER (%) | | | | | |
| --- | --- | --- | --- | --- | --- | --- | --- |
| Year |  | LR | ENR | RFR | GBR | SVR | XGBR |
| 2021 |  | 27.76 | 23.00 | 9.03 | 7.57 | 12.14 | 6.54 |
| 2022 |  | 10.64 | 9.21 | 16.78 | 12.78 | 20.66 | 11.36 |
| 2023 |  | 28.37 | 17.90 | 19.98 | 18.97 | 26.10 | 20.30 |

| LOS |  | ER (%) | | | | | |
| --- | --- | --- | --- | --- | --- | --- | --- |
| Year |  | LR | ENR | RFR | GBR | SVR | XGBR |
| 2021 |  | 57.94 | 57.20 | 14.04 | 15.87 | 11.33 | 1.53 |
| 2022 |  | 6.47 | 5.83 | 8.67 | 13.37 | 3.61 | 8.75 |
| 2023 |  | 28.10 | 28.16 | 28.08 | 28.85 | 17.18 | 28.67 |

| HC |  | ER (%) | | | | | |
| --- | --- | --- | --- | --- | --- | --- | --- |
| Year |  | LR | ENR | RFR | GBR | SVR | XGBR |
| 2021 |  | 35.15 | 11.41 | 5.61 | 14.20 | 12.40 | 18.80 |
| 2022 |  | 4.05 | 25.97 | 13.19 | 8.77 | 12.94 | 12.90 |
| 2023 |  | 31.93 | 9.46 | 0.32 | 3.78 | 3.05 | 8.46 |

**Supplementary table 11. Characteristics used to predict hospitalization burden.**

| Number | Characteristics | HF | LOS | HC |
| --- | --- | --- | --- | --- |
| 1 | Total expenditure | √ | √ | × |
| 2 | Average expenditure | √ | √ | × |
| 3 | Average LOS | √ | × | √ |
| 4 | HF | × | √ | √ |
| 5 | Female | √ | √ | √ |
| 6 | Male | √ | √ | √ |
| 7 | Average age | √ | √ | √ |
| 8 | Average self-paying | √ | √ | √ |
| 9 | Average medical insurance | √ | √ | √ |
| 10 | EF | √ | √ | √ |
| 11 | Admitting diagnosis-A1 | √ | √ | √ |
| 12 | Admitting diagnosis-A2 | √ | √ | √ |
| 13 | Admitting diagnosis-A3 | √ | √ | √ |
| 14 | Admitting diagnosis-A4 | √ | √ | √ |
| 15 | Admitting diagnosis-A5 | √ | √ | √ |
| 16 | Admitting diagnosis-A6 | √ | √ | √ |
| 17 | Admitting diagnosis-A7 | √ | √ | √ |
| 18 | Admitting diagnosis-A8 | √ | √ | √ |
| 19 | Admitting diagnosis-A9 | √ | √ | √ |
| 20 | Admitting diagnosis-A10 | √ | √ | √ |
| 21 | Admitting diagnosis-A11 | √ | √ | √ |
| 22 | Admitting diagnosis-A12 | √ | √ | √ |
| 23 | Discharge diagnosis-other | √ | √ | √ |
| 24 | Discharge diagnosis-improve | √ | √ | √ |
| 25 | Discharge diagnosis-death | √ | √ | √ |
| 26 | Discharge diagnosis-cure | √ | √ | √ |
| 27 | Discharge diagnosis- stabilization | √ | √ | √ |

**Supplementary table 12. Error rates (ERs) for time sequence models.**

| HF (2021) |  | ER (%) | | |
| --- | --- | --- | --- | --- |
| Model |  | None | EF | EF and seasons |
| AR |  | 13.94 | 11.00 | NA |
| SMA |  | 9.96 | 3.75 | 9.97 |
| WMA |  | 7.22 | 6.03 | 0.30 |
| SES |  | 12.22 | 2.24 | 2.19 |
| HLTM |  | 2.27 | 0.86 | 7.35 |
| SARIMAX |  | 9.60 | 5.61 | 0.60 |
| LSTM |  | 12.25 | 11.52 | 2.41 |

| HF (2022) |  | ER (%) | | |
| --- | --- | --- | --- | --- |
| Model |  | None | EF | EF and seasons |
| AR |  | 18.83 | 13.46 | NA |
| SMA |  | 17.09 | 6.95 | 9.70 |
| WMA |  | 15.06 | 15.85 | 8.56 |
| SES |  | 15.33 | 0.89 | 0.11 |
| HLTM |  | 9.84 | 1.94 | 1.77 |
| SARIMAX |  | 17.18 | 16.74 | 4.22 |
| LSTM |  | 18.03 | 11.62 | 2.73 |

| HF (2023) |  | ER (%) | | |
| --- | --- | --- | --- | --- |
| Model |  | None | EF | EF and seasons |
| AR |  | 14.30 | 20.07 | NA |
| SMA |  | 19.90 | 14.49 | 9.76 |
| WMA |  | 19.60 | 18.00 | 9.83 |
| SES |  | 9.70 | 9.34 | 6.12 |
| HLTM |  | 7.24 | 3.34 | 0.23 |
| SARIMAX |  | 16.76 | 7.81 | 0.61 |
| LSTM |  | 20.63 | 6.46 | 3.47 |
